# Exosome tropism and various pathways in lung cancer metastasis

**DOI:** 10.3389/fimmu.2025.1517495

**Published:** 2025-02-14

**Authors:** Hui Chen, Lin Liu, Gang Xing, Dan Zhang, Niumuqie A., Jianlin Huang, Yaling Li, Ge Zhao, Minghua Liu

**Affiliations:** ^1^ School of Pharmacy, Southwest Medical University, Luzhou, China; ^2^ Department of Drug Dispensing, The Third Hospital of Mianyang, Sichuan Mental Health Center, MianYang, China; ^3^ Department of Pharmacy, Luzhou Naxi District People’s Hospital, Luzhou, China; ^4^ Department of Pharmacy, The Affiliated Hospital of Southwest Medical University, Luzhou, China

**Keywords:** lung cancer, exosomes, metastasis, biomarkers, pre-metastatic niche

## Abstract

Lung cancer, characterized by its high morbidity and mortality rates, has the capability to metastasize to various organs, thereby amplifying its detrimental impact and fatality. The metastasis of lung cancer is a complex biological phenomenon involving numerous physiological transformations. Exosomes, small membranous vesicles enriched with biologically active components, are pivotal in mediating intercellular communication and regulating physiological functions due to their specificity and stability. Extensive research has elucidated the production and functions of exosomes in cancer contexts. Multitude of evidence demonstrates a strong association between lung cancer metastasis and exosomes. Additionally, the concept of the pre-metastatic niche is crucial in the metastatic process facilitated by exosomes. This review emphasizes the role of exosomes in mediating lung cancer metastasis and their impact on the disease’s development and the progression to other tissues. Furthermore, it explores the potential of exosomes as biomarkers for lung cancer metastasis, offering significant insights for future clinical advancements.

## 1 Introduction

Based on the estimates of the International Agency for Research on Cancer in 2022, Lung cancer remains one of the most prevalent malignancies globally, causing approximately 1.8 million deaths each year and thus imposing a significant burden on public health (1). It primarily manifests as small cell lung cancer (SCLC) or non-small cell lung cancer (NSCLC) (2), with the latter accounting for roughly 80% of all lung cancer diagnoses (3, 4). An expanding range of therapeutic options now exists for both SCLC and NSCLC, encompassing surgery, radiotherapy, chemotherapy, immunotherapy, and, specifically for NSCLC, treatments such as targeted therapies, antibody-drug conjugates, and bispecific antibodies (5, 6). Treatment strategies are typically tailored and adjusted in response to the patient’s disease progression; for instance, early-stage NSCLC (stage I or II) is primarily managed through tumor resection and adjuvant therapy, whereas advanced stages (III or IV) necessitate chemotherapy or radiotherapy (7).

Despite notable advancements in cancer research, significant improvements in overall survival and quality of life for patients with lung cancer remain elusive (8). The most detrimental aspect of lung cancer is its propensity for metastasis, where malignant cells spread from the lungs to distant organs (9–11).

Metastasis constitutes a defining characteristic of cancer progression (12). Extensive research underscores the pivotal role of the tumor microenvironment (TME) in facilitating metastasis, impacting both the invasive expansion at the primary site and the colonization of distant tissues (13, 14). Paget’s widely endorsed “seed and soil” theory suggests that specific tumor cells adapt to and thrive within the microenvironments of particular organs, facilitating their migration and growth (15). Unlike primary tumors, which often respond well to localized treatments such as surgery or radiotherapy, metastatic cancer is a systemic condition involving multiple organs, making it considerably more challenging to treat (16). Despite advancements in NSCLC management, particularly through chemotherapy, radiotherapy, and surgery, the prognosis for metastatic NSCLC remains dire, especially in cases of metastasis (11). Metastases are implicated in over 70% of NSCLC-related deaths, with the median survival for advanced-stage patients being approximately 18 months post-diagnosis (17).

The concept of the pre-metastatic niche (PMN) refers to the dynamic interaction between metastatic tumor cells and their microenvironment, especially the crosstalk between tumor cells and stromal cells within the target organ (18). The PMN fosters metastasis by promoting neovascularization, enhancing vascular permeability, suppressing immune responses, triggering inflammation, inducing lymphangiogenesis, increasing organ aggregation, and reprogramming cellular functions (10, 19, 20). For instance, miR-29a-3p, embedded in tumor-derived exosomes or liposomal nanopreparations, reduces collagen I levels in lung fibroblasts, thereby disrupting the metastasis-promoting PMN and inhibiting lung cancer metastasis (21). Similarly, Lin28B expression in primary tumors facilitates neutrophil recruitment and polarization towards the N2 phenotype, thereby impairing T-cell function and shaping the immune microenvironment in metastatic settings (22). Moreover, exosomal miR-3157-3p influences the expression of vascular endothelial growth factor (VEGF), matrix metalloproteinases (MMP)-2, MMP9, and occludin in endothelial cells *via* the TIMP/KLF2 axis, driving angiogenesis, increasing vascular permeability, and promoting NSCLC metastasis through the establishment of pre-metastatic ecological sites (23). Additionally, breast cancer-derived exosomes containing Caveolin-1 (Cav-1) modulate inflammatory gene expression in lung epithelial cells, regulating matrix deposition in lung fibroblasts. These exosomes inhibit the PTEN/CCL2/VEGF-A signaling cascade in lung macrophages, thereby fostering M2 macrophage polarization, neovascularization, and PMN formation, ultimately contributing to lung metastasis (24). In summary, the PMN plays an integral role in facilitating metastasis.

Exosomes represent a subtype of extracellular vesicles (EVs), with exosomes being the smaller class and microvesicles constituting the larger variant (25, 26). These vesicles, typically ranging from 30 to 150 nanometers in size, are enclosed by a single lipid bilayer (27, 28). Exosomes are secreted by numerous cell types and are found abundantly in body fluids such as blood, saliva, urine, and milk (27, 29). Extensive research has established exosomes as critical mediators of intercellular communication, facilitating essential biological processes such as angiogenesis, cell proliferation, and metastasis (27, 30–32). They carry a diverse range of bioactive molecules, including proteins, lipids, and nucleic acids, which enable signal transduction or intercellular crosstalk, particularly within the TME (29–31). Notably, exosomal transfer of microRNAs (miRNAs) has emerged as a key mechanism for gene regulation between cells (33). For instance, circular RNAs (circRNAs) act as “sponges” for miRNAs, thereby influencing post-transcriptional gene regulation (34). Furthermore, long non-coding RNAs (lncRNAs) have been identified as key modulators in various cancers (35).

PMN, characterized by enhanced angiogenesis and increased vascular permeability, creates a favorable environment for tumor cell colonization and subsequent metastasis (10, 36). Tumor cells actively modulate distant organs to prepare potential metastatic sites, initiating microenvironmental changes even before metastasis occurs (10, 20). Evidence indicates that both carcinogenesis and PMN formation are heavily influenced by tumor cell-derived exosomes (37). These vesicles are integral to all stages of tumorigenesis and cancer progression, including tumor initiation, growth, metastasis, resistance to therapy, and immune system evasion (37). Additionally, exosomes enhance vascular permeability and trigger inflammatory responses in target tissues, which collectively contribute to the establishment of a conducive pre-metastatic microenvironment (38). These interlinked mechanisms facilitate the formation of metastatic tumors within the target organs (38, 39).

**Figure 1:**
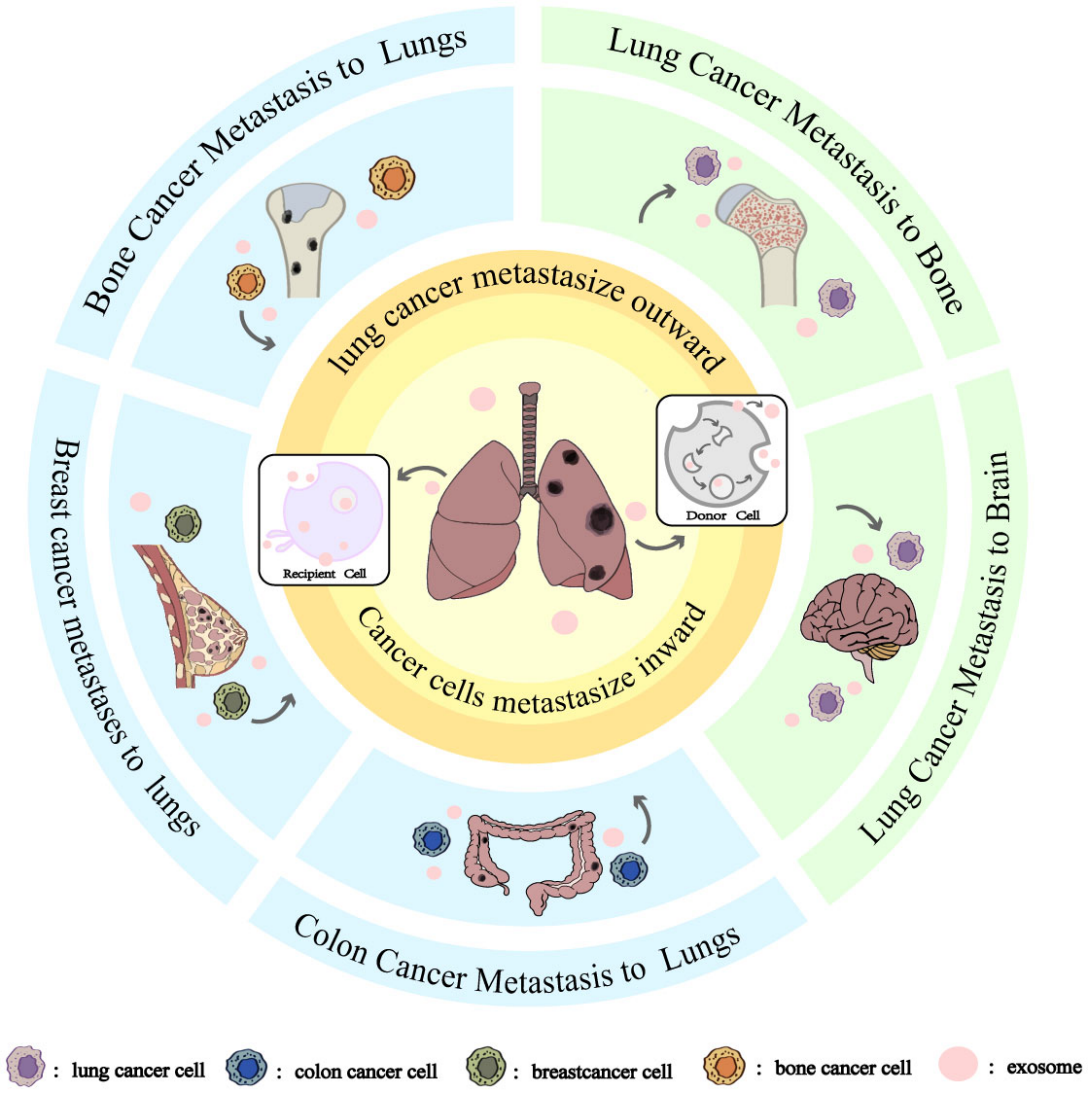
Exosomes and Directional Tissue Orientation in Lung Cancer Metastasis. The blue areas is cancer cells metastasize inward, From top to bottom are bone cancer metastasize to lungs, breast cancer metastasize to lungs, and colon cancer metastasize to lungs. The green area is cancer cells metastasize outward, From top to bottom are lung cancer metastasis to bone and lung cancer metastasis to brain. Whether it is inward or outward metastasis of cancer cells, the metastasis is accomplished through the secretion of exosomes by the donor cells and then diffusion to the recipient cells.

**Figure 2:**
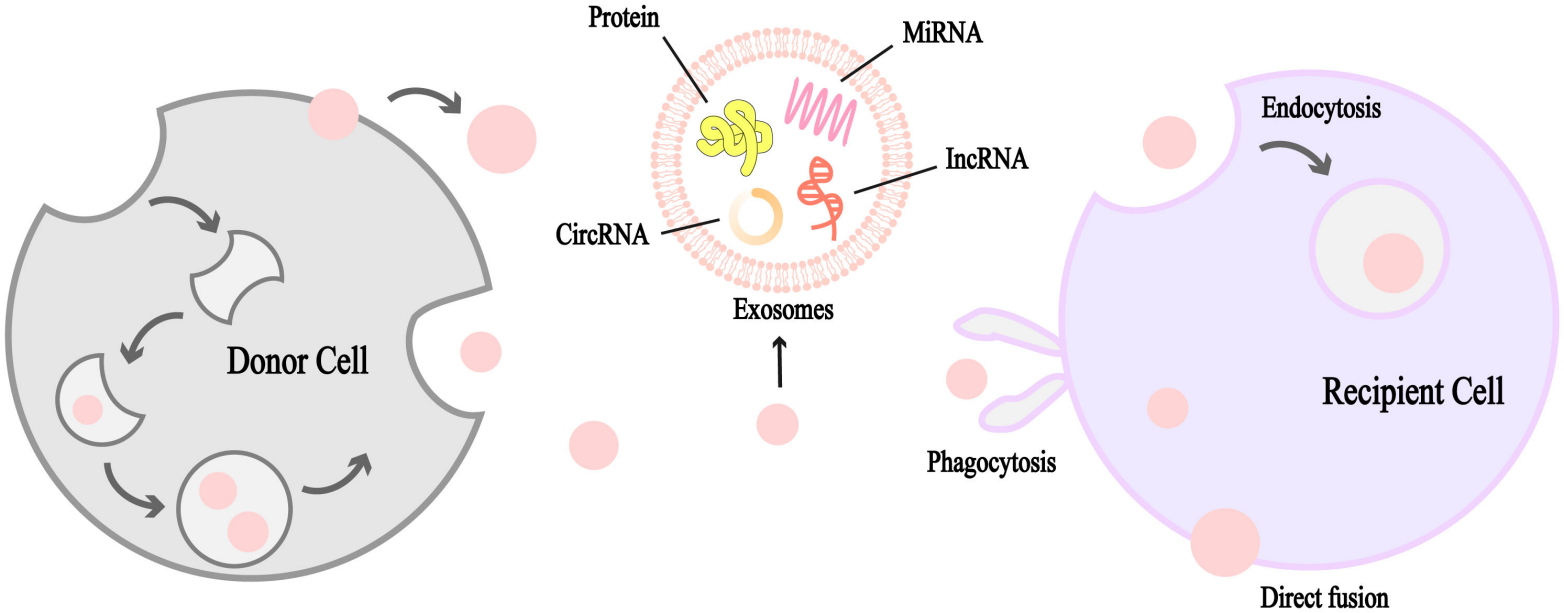
Schematic diagram of the formation and structure of exosomes. Exosomes are unilamellar lipid bilayer vesicles ranging from 30 to 150 nm in diameter containing a variety of biologically active molecules such as miRNAs, circRNAs, IncRNAs, proteins, and other vectors, which mediate intercellular communication, induce signaling, or mediate inter-cellular crosstalk between the cell and the microenvironment in a wide variety of malignant tumors. Exosomes are produced by donor cells and diffuse through various channels to recipient cells, which can receive them by phagocytosis, direct fusion and endocytosis. It is an important medium of intercellular communication.

**Figure 3:**
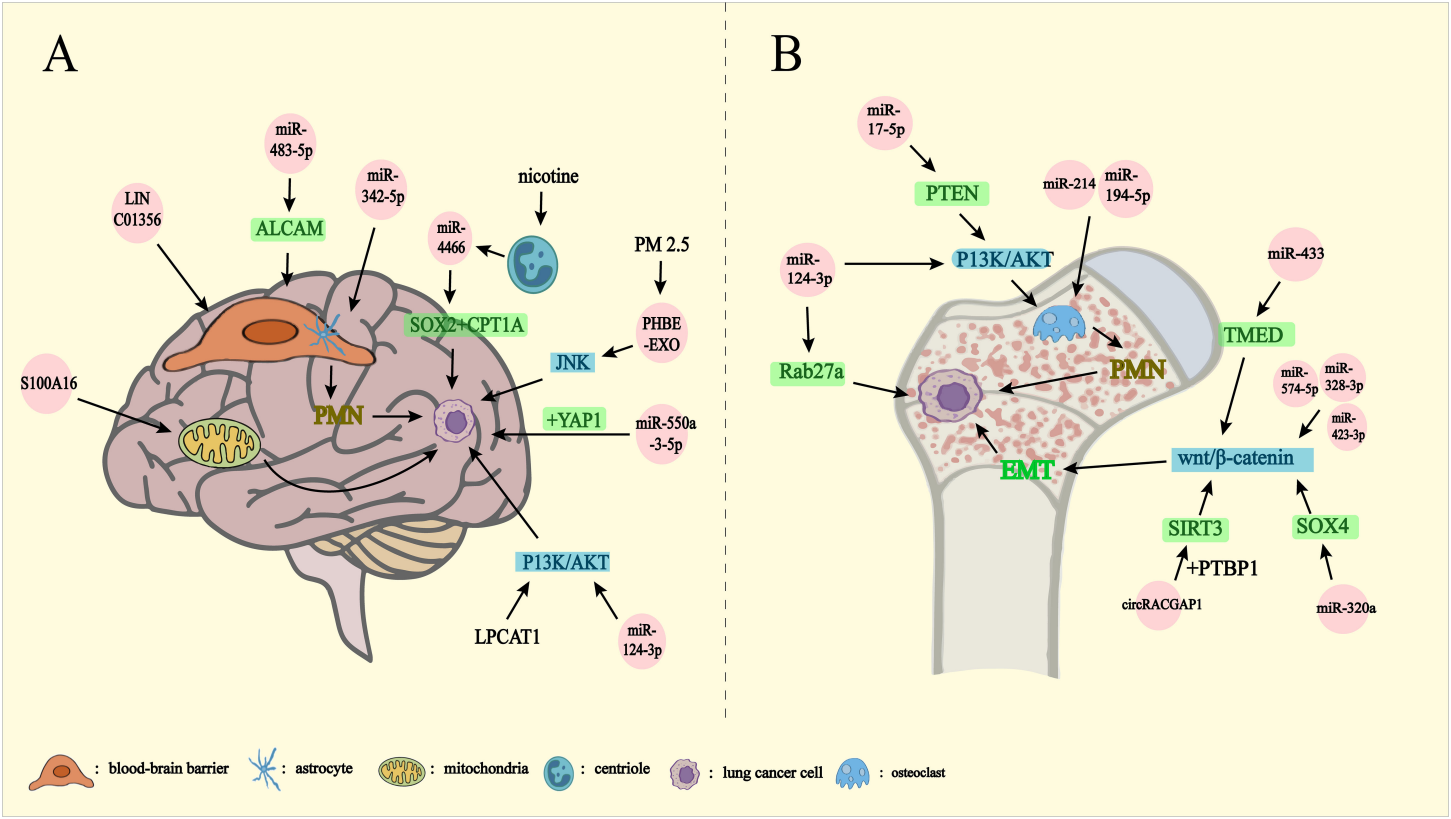
Lung cancer metastasizes outward through exosomes. PMN: pre-metastatic niche EMT: epithelial-mesenchymal transition. Exosomes mediate lung cancer metastasis to brain **(A)**, bone **(B)**. **(A)** miRNA-342-5p, miRNA-483-5p, and LINC01356 affect the BBB on the formation of PMN in the brain by regulating the differentiation of astrocytes, inhibiting the expression of ALCAM, and mediating the remodeling of the blood-brain barrier, respectively. S100A16 controls brain metastasis by up-regulating mitochondrial function and miRNA-550-3-5p binding to YAP1. Chronic nicotine leads to the release of miRNA-4466 from N2-neutrophils thereby activating SOX2, CPT1A genes. PM2.5-treated human bronchial epithelial cells activate the JNK signaling pathway, and miRNA-124-3p, LPCAT1, affects brain metastasis of lung cancer by influencing the P13K/AKT signaling pathway. **(B)** miRNA-214, miRNA-194-5p promote osteoclast formation affecting PMN formation. miRNA-17-5p activates the P13K/AKT signaling pathway via PTEN thereby driving osteoclast formation to promote PMN formation. miRNA-124-3p affects lung cancer bone metastasis by affecting the P13K/AKT signaling pathway and blocking Rab27a protein synthesis. miRNA-433 affects the Wnt/β-catenin signaling pathway through TMED5. miRNA-320a affects the Wnt/β-catenin signaling pathway via SOX4. CircRACGAP1 binding to PTBP1 enhances SIRT3 stabilization affecting the Wnt/β-catenin signaling pathway. miRNA-574-5p, miRNA-328-3p, and miRNA-423-3p affect the Wnt/β-catenin signaling pathway and thus drive the EMT, which affects lung cancer bone metastasis.

**Figure 4:**
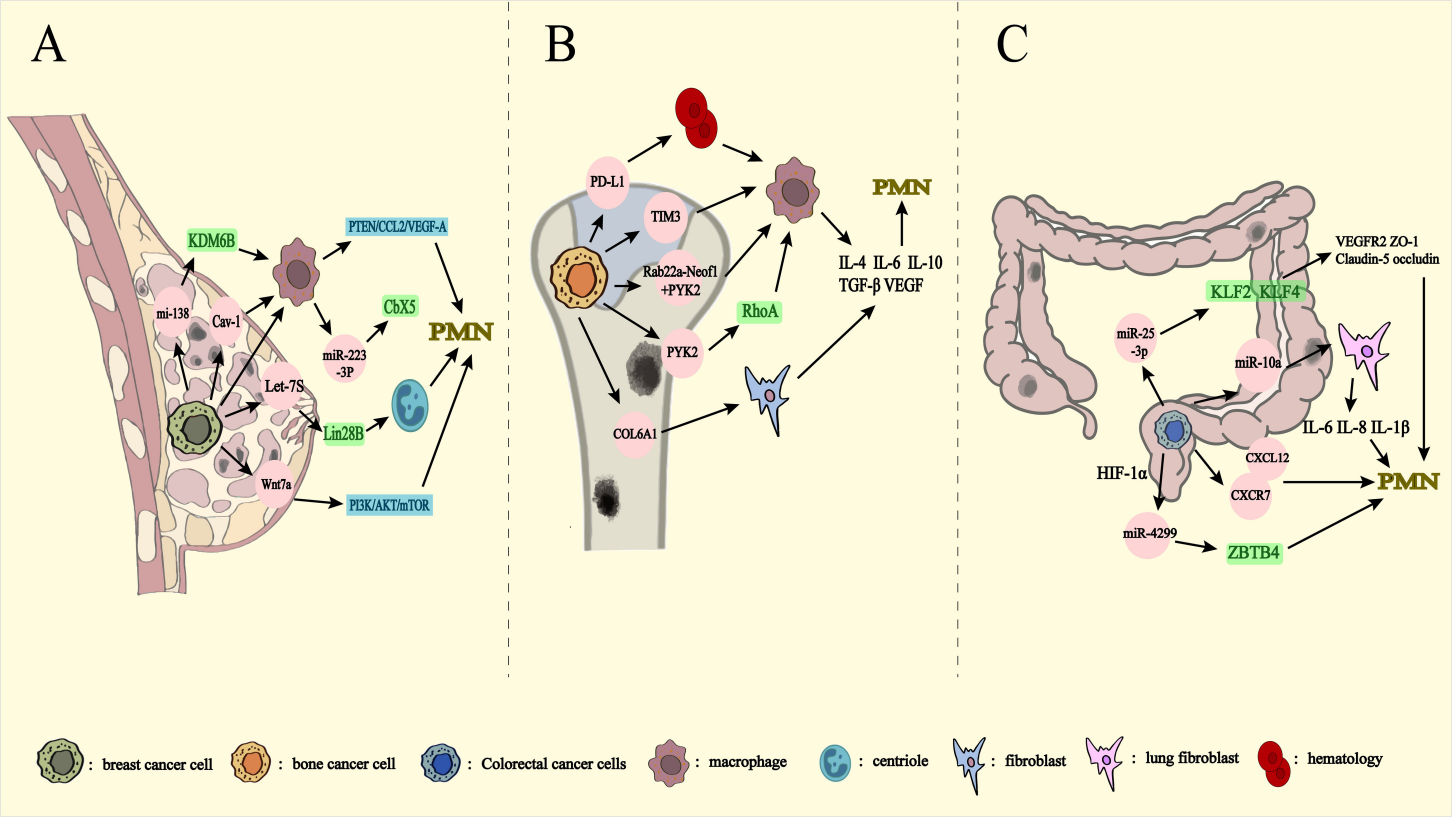
Metastasis from other organs to lungs via exosomes. PMN: pre-metastatic niche. Breast **(A)**, bone **(B)**, and colorectal **(C)** mediate exosome transfer to the lungs. **(A)** miRNA-138-5p decreases the expression level of KDM6B affecting macrophage polarization. Cav-1 inhibition of the PTEN/CCL2/VEGF-A signaling pathway affects macrophage polarization and PMN formation. Let-7s drive the transformation of N2 neutrophils through Lin28B Wnt7a has an impact on PMN formation by activating PI3K/Akt/mTOR, leading to changes in the cytokine milieu of the lung prior to metastasis. Release of miRNA-223-3p by breast cancer cells via macrophage exosomes affects breast cancer lung metastasis by inhibiting Cbx5. **(B)** PD-L1 is released into the bloodstream as exosomes to induce macrophage polarization. Exosome-mediated Tim3 induces macrophage polarization. Rab22a-NeoF1 binds to PYK2, while PYK2 activates the RhoA signaling pathway to promote macrophage polarization. COL6A1 activates fibroblasts. They promotes bone cancer lung metastasis by secreting cytokines such as IL-4, IL-6, IL-10, TGF-β and VEGF. **(C)** miRNA-10a inhibits human lung fibroblasts and reduces the expression of IL-6, IL-8, and IL-1β, affecting PMN production. miRNA-25-3p reduces the expression of VEGFR2, ZO-1, occludin and Claudin5 through KLF2 and KLF4, and affects PMN generation. CXCL12 and CXCR7 are important components of PMN. miRNA-4299 increases expression levels through HIF-1α and promotes colorectal cancer lung metastasis through ZBTB4.

Lung cancer metastasis is a complex, multi-phase process encompassing tumor growth, cell migration, invasion, and neovascularization, all of which ultimately contribute to the development of distant metastases (40). This metastatic cascade can be significantly influenced by exosome-mediated signaling pathways (41). As illustrated in **Figures 1**, **2**, exosomes can be produced by donor cells and transferred and diffused to recipient cells through various pathways to accomplish intercellular communication. lung cancer metastasis can be broadly categorized into two processes: outward and inward. Outward metastasis refers to the dissemination of lung cancer cells to distant organs *via* exosomes, whereas inward metastasis denotes the spread of cancerous cells to the lungs from other primary sites, facilitated by various exosome-driven mechanisms (42). **Figures 3**, **4** highlight that the brain and bones are the predominant targets of outward lung cancer metastasis, while inward metastasis to the lungs commonly originates from breast, bone, colon, and other cancer types (43–47). This review delves into the diverse pathways through which lung cancer metastasizes to distant organs and how other cancers metastasize to the lungs.

## 2 Tissue orientation between exosomes and lung cancer-related metastasis

### 2.1 Outward metastasis of lung cancer

#### 2.1.1 Exosome-mediated pathway of brain metastasis in lung cancer

Brain metastasis (BM) is a frequent and detrimental outcome of lung cancer, significantly impairing treatment efficacy and reducing overall survival (48). Notably, SCLC is recognized as the most aggressive histological subtype, with a pronounced tendency for early BM development (43). BM also constitutes a critical cause of mortality in patients with NSCLC (49). In advanced lung adenocarcinoma (LUAD), the incidence of BM has been reported to reach up to 45% (50). Exosomes are deeply implicated in this process through various mechanisms (**Figure 3A**).

The establishment of a brain PMN in lung cancer individuals with BM largely depends on the disruption of the blood-brain barrier (BBB). Astrocytes, as a critical component of the BBB, play a pivotal role in maintaining the homeostasis of the brain microenvironment (51). Exosomes are capable of crossing the BBB, thereby participating in intercellular communication within the central nervous system (CNS), supporting neuronal development, and modulating inflammatory responses (52). For example, exosomes secreted by the lung cancer cell line H1299, containing MAP2K1, TUBA1C, RELA, and CASP6, induce astrocyte apoptosis and trigger the release of pro-inflammatory cytokines, thereby fostering an immune-evading microenvironment (51).

Exosomal miRNAs play diverse and critical roles in the regulation of BM. In particular, serum-derived exosomal miRNAs from lung cancer individuals with BM are involved in several regulatory pathways. miRNA-342-5p, for instance, has been identified as a downstream target of Notch signaling, promoting the differentiation of neural stem cells into neural progenitors and astrocytes. Meanwhile, the overexpression of miRNA-483-5p has been shown to inhibit ALCAM, a key protein in maintaining BBB integrity (53). Furthermore, miR-550a-3-5p, enriched in exosomes isolated from the plasma of lung cancer individuals with BM, inhibits cell viability and migration while inducing apoptosis in human brain microvascular endothelial cells through YAP1 targeting (54). Additional studies on plasma-derived tumor exosomes from patients with cerebellar metastases have identified six upregulated miRNAs, including has-miRNA-331-5p, -542-5p, -26a-2-3p, -99a-3p, -184, and -3065-5p, which may serve as potential biomarkers for BM (48). Interestingly, miR-124-3p targets the 3’UTR of Rab27a, reducing exosome secretion and inhibiting the PI3K/AKT signaling pathway, thereby curtailing NSCLC metastasis (55). Moreover, lysophosphatidylcholine acyltransferase 1 (LPCAT1), an enzyme involved in phospholipid metabolism, has been found to regulate the PI3K/AKT pathway by modulating the MYC transcription factor, thus enhancing cancer cell growth and metastasis (56). Single-cell RNA sequencing of LUAD brain metastases confirmed significantly elevated LPCAT1 expression in lung cancer cells at BM sites compared to primary lung tumor cells (49). Upregulation of LPCAT1 in NSCLC has been associated with an increased incidence of BM, while LPCAT1 inhibition effectively curbs lung cancer cell proliferation and metastasis (49, 56).

LncRNAs and proteins in exosomes are equally pivotal in the development of brain metastasis (BM). For example, exosomes secreted by the NSCLC cell line H1299 that carry LINC01356 play a pivotal role in remodeling the BBB and facilitating BM in lung cancer (57). Additionally, calcium-dependent proteins and S100 proteins are strongly linked to BM formation (58). Notably, exosome-mediated delivery of S100A16 from brain microvascular endothelial cells enhances SCLC cell survival and promotes BM by boosting mitochondrial function (43). Moreover, plasma exosomal MUC5B, a cell surface glycoprotein that stimulates brain metastasis, has shown potential as a biomarker for diagnosing BM in patients with lung cancer (58).

The influence of external environmental factors on lung cancer brain metastasis is also profound. Studies indicate that patients with lung cancer with a history of smoking are more susceptible to BM compared to non-smokers. Chronic nicotine exposure leads to the accumulation of N2-neutrophils around brain metastases, which release exosomes carrying miRNA-4466. These exosomes activate tumor stem cell-associated genes and metabolic regulators, such as SOX2 and CPT1A, thereby promoting brain tumor metastasis and solid tumor growth (59). Similarly, exosomes derived from human bronchial epithelial cells exposed to PM2.5 have been shown to enhance the migration, invasion, and epithelial-mesenchymal transition (EMT) of lung cancer cells, activating the JNK signaling pathway to accelerate lung cancer metastasis *in vivo* (60).

In summary, the progression of brain metastasis during lung cancer metastasis to the brain, mediated by exosomes, is shaped by both intrinsic and extrinsic environmental factors. The establishment of the PMN within the intrinsic environment further contributes to BM development. However, safeguarding the BBB is paramount in preventing tumor metastasis to the brain. Therefore, future research into strategies aimed at enhancing the protective function of the BBB during tumor cell metastasis holds significant promise.

#### 2.1.2 Exosome-mediated pathway of bone metastasis in lung cancer

The skeletal environment plays a pivotal role in supporting lung cancer cell proliferation (61). Bone is one of the most frequent sites for lung cancer metastasis, with an incidence rate of 30-40% among patients with lung cancer (44). The presence of bone metastases drastically reduces the three-year survival rate from 71.6% to 46.8% (62). Bone metastases in lung cancer are classified as either osteoblast-associated or osteoclast-associated. In metastatic NSCLC, osteolytic bone destruction driven by heightened osteoclast activity is the predominant mechanism (63). Exosomes are deeply involved in this pathological process (**Figure 3B**), delivering bioactive molecules—such as proteins, lipids, and non-coding RNAs (ncRNAs)—that play a critical role in bone remodeling by modulating osteoblast function (64).

The interaction between osteoclasts and cancer cells is reciprocal. Exosomes carrying miR-214 and miR-194-5p secreted by lung cancer cells promote osteoclast formation and enhance bone resorption, creating a bone marrow microenvironment conducive to cancer cell survival and metastasis (65, 66). Exosomal miRNAs play a pivotal role in facilitating lung cancer bone metastasis by modulating key signaling pathways. Research suggests that the dysregulation of miRNAs, particularly those involved in the Wnt/β-catenin pathway, significantly drives cancer progression, metastatic spread, and resistance to therapy (67). This pathway is integral to the EMT, a critical process in tumor development and metastasis, and is especially important in CNS maturation (68, 69). Key miRNAs, such as miR-574-5p, miR-328-3p, miR-423-3p, miR-770, miR-433, and miR-1260b, actively participate in the Wnt/β-catenin pathway, influencing EMT. Notably, inhibiting miR-574-5p, miR-328-3p, and miR-423-3p may synergistically disrupt the Wnt/β-catenin signaling pathway, potentially playing a critical role in preventing lung cancer bone metastasis. Further analysis has identified 43 miRNAs, predominantly located on chromosome 14, that are upregulated in bone metastases and linked to EMT and bone metastasis (69). For instance, miR-433 targets the 3′-UTR of TMED5, thereby inhibiting the Wnt/β-catenin pathway (70), while miR-320a from human umbilical cord MSC-derived exosomes modulates this pathway by targeting SOX4 (71). Moreover, circRACGAP1 enhances the stemness and metastatic potential of NSCLC cells by interacting with PTBP1, stabilizing SIRT3, and activating the RIF1-mediated Wnt/β-catenin pathway (72).

The PI3K/AKT signaling pathway also plays a central role in lung cancer bone metastasis. miR-17-5p promotes osteoclast formation by targeting the tumor suppressor PTEN and activating the PI3K/AKT signaling cascade in lung cancer bone metastasis (73). Conversely, miR-124-3p inhibits PI3K/AKT signaling by targeting the 3’UTR of Rab27a, reducing exosome secretion and thereby limiting NSCLC metastasis (55).

Bone marrow mesenchymal stem cells (BMSCs) also contribute to the proliferation and further metastasis of lung cancer cells in the bone marrow microenvironment. Quiescent lung cancer cells release exosomes that are internalized by BMSCs, enhancing the glycolytic capacity of BMSCs *via* the insulin-like growth factor 1 receptor (IGF-1R) signaling pathway. Blocking IGF-1R signaling or glycolytic pathways effectively slows the proliferation of lung cancer cells within the bone marrow (61).

Both the Wnt/β-catenin and PI3K/AKT signaling pathways play integral roles in lung cancer metastasis to bone *via* exosomes. By modulating the bone marrow microenvironment, these pathways provide cancer cells with enhanced survival and proliferation capacities, ultimately promoting metastasis. The mutual reinforcement between osteoclasts and cancer cells underscores the importance of osteoclasts in the metastatic process. Future research should focus on understanding osteoclast formation and function, as altering their properties could potentially disrupt the metastatic cycle.

In conclusion, exosomes play a critical role in driving the metastasis of lung cancer to the brain and bone through various molecular mechanisms, making them a promising target for future diagnostic and therapeutic interventions in the management of this lethal disease.

### 2.2 Inward metastasis to the lungs from other sites

#### 2.2.1 Exosome-mediated pathway of lung metastasis in breast cancer

Breast cancer (BC) metastasis accounts for approximately 90% of fatalities associated with the disease, with lung metastasis being a predominant route (45). The 5-year survival rate for patients with metastatic breast cancer is a mere 20% (74), and it is estimated that 60-70% of those with lung metastases eventually succumb to recurrence (75). Exosomes play a pivotal role in facilitating these metastatic processes (**Figure 4A**).

Exosome-mediated PMN formation is essential for the metastasis of breast cancer cells to the lungs. Exosomes derived from metastatic cells are integral in creating a microenvironment conducive to metastasis, particularly through the involvement of specific proteins (76). For instance, exosomes from BC cells containing Cav-1 upregulate inflammatory gene expression in lung epithelial cells, thereby modulating matrix deposition in lung fibroblasts. These exosomes disrupt the PTEN/CCL2/VEGF-A signaling pathway in lung macrophages, promoting M2-type polarization, neovascularization, and PMN formation, all of which facilitate lung metastasis in breast cancer (24). Moreover, exosomes containing Wnt7a protein from low-metastatic cell lines, such as LM.4T1, can enhance the metastatic potential of high-metastatic cells by activating the PI3K/AKT/mTOR signaling pathway. These low-metastatic cells also stimulate angiogenesis within the TME, providing a more favorable setting for highly metastatic cells to evade immune surveillance. Notably, Wnt7a increases the risk of lung metastasis in triple-negative breast cancer, highlighting its potential as a novel therapeutic target (77).

High levels of the lncRNA Lin28B, coupled with low levels of let-7s, are prognostic indicators for poor outcomes and lung metastasis in patients with breast cancer. Lin28B alters the cytokine environment in the lungs before metastasis occurs, facilitating neutrophil recruitment and N2 polarization. Even in the absence of tumor cell migration, Lin28B triggers changes in the PMN, leading to the production of IL-6 and IL-10, which drive the N2-type conversion (22). Comparative analysis of primary tumors, non-tumor tissues, and lung metastases has shown that tumor suppressors miR-200c and let-7a are downregulated in both tumors and metastatic tissues, but their levels in exosomes are elevated, particularly in lung metastases. Exosomal miR-200c may suppress the immune response of F4/80+ macrophages, negating the tumor-suppressive effects on recipient cells. Rab1A facilitates the packaging of miR-200c into exosomes, circumventing its inhibitory effect on tumor proliferation. Thus, targeting anti-Rab1A proteins to enhance miR-200c expression could impede breast cancer metastasis to the lungs (78).

miRNAs within exosomes secreted by breast cancer cells also influence macrophage remodeling and programming. For example, miR-138-5p translocates into macrophages and downregulates KDM6B expression, regulating macrophage polarization (79). M2-type macrophages, in particular, play a tumor-supportive role, aiding in tumor invasion and metastasis (80, 81). Exosomes derived from tumor-associated macrophages (TAMs), containing miR-223-3p, promote lung metastasis by targeting and inhibiting Cbx5, thus facilitating breast cancer cell metastasis to the lungs (82).

In conclusion, breast cancer utilizes exosomes to influence target genes and proteins, modify multiple signaling pathways, and reshape macrophage polarization. These changes ultimately contribute to the formation of the PMN, which drives the initiation of lung metastasis in breast cancer cells.

#### 2.2.2 Exosome-mediated pathway of lung metastasis in osteosarcoma

Osteosarcoma (OS) predominantly arises in regions of rapid bone growth, such as the femur, tibia, and humerus. This malignancy is characterized by cancer cells that produce immature bone or bone-like tissue (83). OS is one of the most common malignant tumors originating in the skeletal system, particularly affecting children and adolescents, and is notorious for its high metastatic potential (84). The 5-year overall survival rate remains relatively low, around 20%, largely due to the presence of lung metastases in 15-30% of patients at the time of diagnosis (85). Exosomes play a critical role in the lung metastasis of bone cancer (**Figure 4B**).

The complex genetic makeup of OS suggests that immune suppression is a key factor in its progression (86). In patients with OS, immunosuppressive cells, supportive cells within the TME, and tumor cells can release programmed death ligand 1 (PD-L1) *via* exosomes, which collectively contribute to systemic immunosuppression (87). Studies have shown that PD-L1 expression in the TME, both in mouse models and humans, promotes systemic immune suppression, thereby increasing tumor burden and reducing patient survival across various cancers (88). In contrast, lung metastasis was significantly reduced in mice treated with exosomes from PD-L1 knockdown cells, suggesting that exosomal PD-L1 is crucial in promoting lung metastasis in OS (89). Furthermore, tumor-derived exosomes enhance glucose uptake *via* the TLR2 and NF-κB pathways, leading to increased nitric oxide synthase 2 (NOS2) levels, which inhibit mitochondrial oxidative phosphorylation. This metabolic shift increases PD-L1 expression by converting more pyruvate to lactate, which in turn stimulates NF-κB activity and polarizes macrophages towards an immunosuppressive phenotype (90).

Macrophages, as primary phagocytes involved in the innate immune response, play a critical role in combating OS and other cancers, significantly influencing survival outcomes (91). M1 macrophages exert tumor-suppressive effects, while M2 macrophages promote tumor growth and metastasis. OS cells enhance migration, invasion, EMT, and lung metastasis primarily through exosome-mediated immunomodulatory factors, such as Tim3, which induce M2 macrophage polarization and the secretion of cytokines like IL-4, IL-6, IL-10, TGF-β, and VEGF (81, 91–94). For example, visfatin treatment of chondrosarcoma cells has been shown to increase exosome production, which in turn stimulates M2 macrophage polarization, enhancing chondrosarcoma motility and contributing to lung metastasis (95). However, interactions between tumor cells and macrophages may vary depending on the metastatic potential of the tumor. For instance, EVs from highly metastatic OS interact differently with macrophages compared to those from less metastatic cells. In highly metastatic K7M2 OS cells, EVs decrease the levels of pro-inflammatory cytokines such as INFγ, IL-2, IL-6, and TNFα in macrophages, suggesting that these EVs further promote M2 macrophage differentiation, reinforcing the metastatic process. This represents the first evidence of differential interactions between OS-EVs and macrophages based on their polarization states (96).

Exosomal proteins are pivotal in facilitating the metastasis of OS to the lungs. One key player in this process is the upregulation of the COL6A1 gene in OS tissues, which has been strongly correlated with an increased likelihood of lung metastasis and reduced survival rates in patients with OS. This upregulation occurs through the binding of the transcription factor c-Jun to p300, which enhances the acetylation modification (H3K27ac) of histone H3 lysine 27 in the promoter region of the COL6A1 gene. Moreover, COL6A1 facilitates OS metastasis by downregulating STAT1 expression through its degradation *via* the ubiquitin-proteasome pathway. OS cells package COL6A1 into exosomes and transfer it to fibroblasts, inducing their activation and stimulating TGF-β secretion, which in turn enhances the invasive and migratory abilities of OS cells (92). Additionally, exosomes derived from OS cells carrying Rab22a-NeoF1 fusion proteins and PYK2 further exacerbate lung metastasis by recruiting bone marrow-derived macrophages to establish a favorable metastatic microenvironment. PYK2 in exosomes activates the RhoA signaling pathway within OS cells and promotes the generation of M2-type macrophages, which are known to support tumor metastasis, thereby accelerating OS metastasis to the lungs (97). Furthermore, Rab27a, a gene critical for exosome-specific protein transport, is essential for OS cell metastasis. Knocking out Rab27a in OS cells reduces exosome secretion, significantly lowering their metastatic potential to the lungs (89).

As discussed, OS cells exploit exosomes to influence several pathways, including macrophage polarization and fibroblast activation, by releasing various cytokines. These exosomal interactions create an immunosuppressive microenvironment that favors metastasis, supporting the migration of OS cells and the development of lung metastasis. Thus, identifying strategies to detect, disrupt, or prevent the formation of this immunosuppressive environment represents a promising avenue for future research. Such approaches may offer new therapeutic insights into mitigating the progression and metastasis of OS to the lungs.

#### 2.2.3 Exosome-mediated pathway of lung metastasis in colorectal cancer

Colorectal cancer (CRC) is the third most common malignancy globally and ranks second in cancer-related mortality (4). Approximately 20-25% of patients with CRC present with synchronous metastatic disease at diagnosis (98), with the liver and lungs being the most frequent sites of metastasis. Approximately 10-25% of patients with CRC eventually develop lung metastases (47). Exosomes play a significant role in mediating CRC lung metastasis through various mechanisms (**Figure 4C**).

The formation of PMN is a critical step in CRC lung metastasis, where exosomal miRNAs facilitate communication between cancer cells and stromal cells (99). Notably, miR-10a expression in CRC-derived exosomes is inversely correlated with the depth of tumor invasion. Exosomal miR-10a from CRC cells inhibits the proliferation and metastatic potential of normal human lung fibroblasts (NHLFs) and reduces the expression of inflammatory cytokines, such as IL-6, IL-8, and IL-1β in NHLFs (100). Additionally, miR-25-3p secreted by CRC cells targets KLF2 and KLF4 in vascular endothelial cells, downregulating the expression of vascular endothelial growth factor receptor 2 (VEGFR2), ZO-1, occludin, and Claudin5, thereby affecting neovascularization and PMN formation, promoting CRC metastasis (36).

CXCL12 is another key component of the PMN that is often upregulated before metastasis occurs. In noncancerous lung tissues of patients with CRC, CXCL12 expression is significantly higher compared to patients with benign lung disease. Immunohistochemistry analysis reveals that CXCR7 and CXCL12, both indicators of colon cancer lung metastasis, are more highly expressed in lung metastatic tissues than in primary tumor sites (101). Moreover, circLONP2 interacts with the DGCR8/Drosha complex in a DDX1-dependent manner, enhancing the maturation of primary microRNA-17. The resulting elevated levels of miR-17-5p can be encapsulated in exosomes and transferred to neighboring cells, thereby promoting migration and spread of CRC cells (102).

Hypoxic conditions in tumors also enhance exosome production in CRC. In a low-oxygen environment, tumor cells alter their metabolic pathways and migrate toward oxygen-rich areas, promoting metastasis (103). For instance, macrophages exposed to exosomes under hypoxic conditions undergo M2-type macrophage differentiation through activation of the AMPK/p38 signaling pathway, which in turn supports lung tumor proliferation and metastasis (104). Hypoxia also elevates miR-4299 levels in CRC cells and hypoxia-derived exosomes in a hypoxia-inducible factor 1 alpha (HIF1α) dependent manner, which directly targets ZBTB4, thereby promoting CRC proliferation and metastasis (103).

Thus, CRC cells utilize mechanisms such as hypoxia and cytokine production to increase exosome release and promote PMN formation, contributing to lung metastasis.

In summary, exosomes play a central role in the metastasis of breast, bone, and colorectal cancers to the lungs by influencing various signaling pathways and facilitating PMN formation. This underscores the importance of exosome-mediated PMNs in metastasis and highlights potential diagnostic and therapeutic targets for mitigating lung metastasis from primary tumors.

## 3 Exosomes influence lung cancer development

### 3.1 Tumor exosomes influence lung cancer metastasis through angiogenesis

In the TME, angiogenesis is a critical driver of cancer progression and metastasis (20, 105). VEGFA and its receptor VEGFR2, are central to the regulation of tumor-induced angiogenesis (106). Exosome-transported miRNAs produced by cancer cells play a vital role in facilitating neovascularization, reshaping the TME, and establishing a PMN (36, 105, 107).

One example is exosome-transported miR-197-3p, which has been shown to downregulate the expression of TIMP3 in human umbilical vein endothelial cells (HUVECs). This downregulation activates VEGFR2, stimulating downstream ERK signaling and further promoting angiogenesis. Simultaneously, miR-197-3p targets TIMP2, reducing the expression of MMP2 and MT1-MMP, thus enhancing neovascularization (108). Similarly, exosomal miR-3157-3p from lung cancer cells downregulates KLF2, ZO-1, occludin, and Claudin5, while simultaneously elevating levels of VEGF, MMP2, and MMP9, significantly increasing the permeability of HUVECs and promoting angiogenesis (23). Additionally, pedunculated vesicles serve as key structural elements in neoangiogenesis, with angiogenesis playing a fundamental role in their formation within the TME (20). For instance, phosphoglycerate translocase 1 (PGAM1) in HUVEC-derived exosomes interacts with γ-actinin (ACTG1) to stimulate the development of pedunculated vesicles, thereby contributing to the neovascularization process (109).

Thus, exosomes significantly influence angiogenesis by modulating VEGF and associated pathways, further promoting cancer metastasis through enhanced blood vessel formation.

### 3.2 Tumor exosomes influence lung cancer metastasis through macrophage polarization

In lung cancer, macrophages are the most abundant type of white blood cell within the TME and play a pivotal role in all stages of cancer development (110). The complex interactions between tumor cells and TAMs significantly impact tumor progression. Tumor cells drive macrophage polarization towards the immunosuppressive M2 phenotype through various mechanistic pathways, which exacerbate tumor malignancy and are closely associated with poor prognosis (111). Tumor cells achieve this by releasing inflammatory cytokines, chemokines, extracellular vesicles, and tumor metabolites, which induce TAMs to adopt the M2 phenotype. In turn, M2-type TAMs enhance tumor cell proliferation and EMT (112) and promote tumor angiogenesis, PMN formation, immune escape, and ultimately tumor growth and metastasis (80, 112, 113). Recent studies have identified key pathways involved in tumor-derived exosome-mediated macrophage transformation, including the NF-κB and STAT3 signaling pathways, as well as hypoxic conditions (90).

#### 3.2.1 NF-κB signaling pathway

Exosomes promote TAMs polarization toward the M2 phenotype by activating the NLRP6/NF-κB signaling pathway (114, 115). Tumor cells also transfer TRIM59 *via* exosomes to macrophages, where it interacts with ABHD5 to activate the NLRP3 signaling pathway. This promotes the release of pro-inflammatory cytokines such as IL-18 and IL-1β, further contributing to an immunosuppressive environment (114, 115). Additionally, tumor cells undergoing enhanced glycolysis, which supports their proliferation, angiogenesis, and metastasis (116), release exosomes that boost glucose uptake through TLR2 and NF-κB pathways. These exosomes increase NOS2 levels, inhibiting mitochondrial oxidative phosphorylation and shifting energy production towards lactate. This metabolic change amplifies NF-κB activity, which upregulates PD-L1 expression and further drives macrophages towards the M2 phenotype, thereby enhancing lung cancer’s metastatic potential (90).

#### 3.2.2 STAT3 signaling pathway

The STAT3 signaling pathway is another critical factor in macrophage polarization. Activation of this pathway leads to M2 macrophage differentiation. Exosomal MFG-E8, a significant component of small cellular debris exosomes, mitigates inflammatory responses by activating SOCS3; however, this also interferes with the regulation of STAT3 phosphorylation, facilitating M2 polarization (117). PYK2 in tumor exosomes can also trigger STAT3 activation in macrophages by activating the RhoA pathway in OS cells (97). Furthermore, exosomal miR-19b-3p from LUAD cells induces M2 macrophage polarization by targeting PTPRD proteins to activate STAT3 signaling, supporting lung metastasis of tumor cells (118).

#### 3.2.3 Hypoxia pathway

Exosomes released under hypoxic conditions further promote M2 macrophage polarization. In NSCLC, circPLEKHM1 in exosomes, regulated by HIF1α, activates the OSMR/JAK/STAT3 signaling pathway, promoting M2 polarization in the TME by enhancing the interaction between PABPC1 and eIF4G (46). Additionally, the AMPK/p38 signaling pathway is upregulated under hypoxia, driving the differentiation of macrophages into the M2 phenotype (104).

#### 3.2.4 Bioactive substances in exosomes affect M2 polarization

Exosomes play a pivotal role in driving macrophage polarization toward the M2 phenotype by transmitting various bioactive molecules. For instance, lung cancer cell-derived exosomes can facilitate this transition by delivering lncRNAs such as PCAT6 and LINC00963, which promote M2 macrophage polarization (119, 120). Additionally, exosomal circ_0001715 internalizes and inhibits miR-205-5p, leading to the upregulation of TREM2, which further stimulates M2 macrophage polarization and creates a feedback loop that accelerates lung cancer growth, metastasis, and EMT (120–122). In contrast, lung cancer cells can also release miR-770-containing exosomes, which suppress M2 macrophage transformation by targeting the MAP3K1 gene, thereby reducing immunosuppression (121). Tumor-derived exosomes additionally enhance I-IFN secretion *via* the circPIK3R3/miR-872-3p/IRF7 axis, boosting the anti-tumor immune response of CD8+ T cells (123).

Beyond tumor-secreted exosomes, M2-type TAMs contribute to NSCLC metastasis by releasing exosomes carrying miRNAs such as miR-155 and miR-196a-5p, which promote metastasis by directly inhibiting the RASSF4 gene through interaction with its 3’UTR (124). Tumor cells induce M2 macrophage polarization *via* multiple pathways, including NF-κB, STAT3, and hypoxia-related mechanisms, thereby intensifying the malignant characteristics of the tumor. M2 macrophage polarization not only enhances metastasis of lung cancer to distant sites through exosomes but also facilitates metastasis from other regions, particularly bone cancer, to the lungs. This expands diagnostic options by broadening the range of potential indicators. Moreover, macrophages can interact with other immunosuppressive cells, tumor cells, and various components within the microenvironment, fostering an immunosuppressive setting during M2 polarization. Targeting this process through artificial interventions aimed at engaging macrophages and disrupting the immunosuppressive milieu during the M2 transition could significantly mitigate cancer metastasis.

### 3.3 Tumor exosomes affect lung cancer metastasis through other pathways

miRNAs play a significant role in influencing tumor progression and prognosis through their regulatory effects on the TME. The release of exosomes is closely linked to the expression levels of various miRNAs (125). As indicated in **Table 1**, exosomal miRNAs exhibit dual roles in lung cancer metastasis, with certain miRNAs promoting cancer progression through multiple pathways. Among the identified promoters of lung cancer metastasis are miR-133a-3p, miR-665, miR-486-5p, miR-375-3p, miR-1260b, miR-3180-3p, miR-210-3p, and miR-582-3p.

**Table 1:** Tumor exosomes affect lung cancer metastasis through various pathways.

Exosomes	Cellular source	Recipient cells	Molecular targets	Possible mechanism	Effects	Reference
Positive regulation of lung cancer						
miR-133a-3p	Serum	A549	SIRT1	↓SIRT1	↑Proliferation, migration, invasion, and neovascularization	(126)
miR-582-3p	A549, H1299	A549 H1299	SFRP1	↓SFRP1	↑Proliferation, migration, and invasion	(127)
miR-665	MPE	H1975 H1650H446	HEYL	Notch signaling pathway	↑Encroachment and migration	(128)
miR-486-5p	Serum and tu-mor tissue	Vascular Endothelial cell	Unknow	CADM1/TJs pathway	↓Vascular endothelial barrier function	(129)
miR-375-3p	H446, H1048	Vascular Endothelial cell	Unknow	↓claudin-1	↓Vascular barrier function ↑Metastasis	(130)
miR-1260b	H1299, A549	Peripheral tumorcells	Unknow	Wnt/β-catenin signaling pathway	↑Neovascularization, migration, and chemotherapy resistance	(131)
	A549, Calu-1	HUVEC	HIPK2	↓HIPK2		(105)
miR-210-3p	A549	A549, NCI-H17-03	FGFRL1	↑N-calmodulin, poikilodulin, MMP-9, MMP-1, ↓E-calmodulin	↑Invasion, migration, EMT, MMP-9, and MMP-1	(132)
circSATB2	Serum	A549, H460, H1299	FSCN1	↑miR-326, ↓FSCN1	↑Proliferation, migration, and invasion	(142)
lncRNA ZBED5-AS1	HCC827	H1299, A549	Unknow	↑ZNF146/ATR/CHK1 signaling, ↑EMT	↑Proliferation, migration, and invasion	(139)
Lin HOTAIR	NCI-H1975	A549	Unknow	Unknow	↑Proliferation and migration	(141)
LINC00963	A549	A549, NCI-H1650	Unknow	↑EMT, ↑Macrophage M2	↑Proliferation, migration, invasion, and EMT	(120)
circ_0008717	NSCLC	A549, H1299	Unknow	↑Expression of PAK2	↑Tumor formation	(143)
circRAPGEF 5	A549, H1299	HCC827, A549, H1299	ZEB1	miR-1236-3p/ZEB1 signaling p-athway	↑Proliferation, migration, invasion, and EMT	(144)
Negative regulation of lung cancer						
miR-124-3p	Serum	A549, NCI-H1299	Rab27a	↓Rab27a ↓PI3K/AKT signaling	↓Migration and infestation	(55)
miR-15a-5p	A549	A549	CDCA4	Regulation of EMT, ↓waveform protein, N-calmodul-in, ↑E-calmodulin expression	↓Proliferation, migration, and infestation	(137)
miR-338-3p	Unknow	A549, SK-MES-1	CHL1	↓MAPK signaling pathway	↓Proliferation and migration	(138)
miR-3180-3p	A549	A549	FOXP4	↓FOXP4, ↑miR-3180-3p	↓Proliferation and migration	(135)
lncRNA FGD5-AS1	A549	A549, H1299	miR-944, MACC1	↓miR-944, ↑MACC1	↓Proliferation, migration, and invasion	(140)

The symbol ↓ means weaken, the symbol ↑ means strengthen.

For example, miR-133a-3p, found in lung cancer serum exosomes, silences specific proteins such as Sirtuin 1 (SIRT1) through targeted mechanisms. Overexpression of miR-133a-3p has been shown to significantly enhance the biological properties of A549 lung cancer cells, promoting cell viability, migration, invasion, angiogenesis, and proliferation (126). Similarly, miR-582-3p in exosomes derived from hypoxic NSCLC cell lines (A549 and H1299) enhances proliferation, metastasis, and invasion of normoxic NSCLC cell lines by inhibiting the expression of secreted frizzled-related protein 1 (SFRP1), thus contributing to tumorigenesis through the modulation of various signaling pathways (127). In another case, exosomes derived from malignant pleural effusion (MPE) in lung cancer, enriched with miR-665, are taken up by NSCLC (H1975, H1650) and SCLC (H446) cells. This uptake stimulates their invasive and metastatic capabilities by modulating the transcription factor HEYL, which acts downstream of the Notch signaling pathway (128). Moreover, miR-486-5p present in serum and tumor tissues of patients with NSCLC promotes metastasis by targeting the CADM1/TJs pathway in vascular endothelial cells, impairing endothelial barrier function and increasing the concentration of miR-486-5p in the bloodstream (129). Similarly, miR-375-3p, released by SCLC cells (H446 and H1048), acts on vascular endothelial cells by downregulating claudin-1, a key tight junction protein, further impairing vascular barrier function and promoting metastasis (130). In another instance, exosomes from H1299 and A549 cells have been observed to deliver miR-1260b to surrounding tumor cells, reducing the expression of SFRP1 and Smad4. This activates the Wnt/β-catenin signaling pathway, enhancing the invasive ability of LAC cells and promoting metastatic progression (131).

miRNAs also target specific proteins or genes to drive tumor progression. miR-1260b enhances neovascularization, migration, and chemotherapy resistance by inhibiting HIPK2 and regulating the Wnt/β-catenin signaling pathway in lung cancer cells (105). Additionally, exosomal miR-210-3p released from lung stem cells derived from A549 cells enhances the migration and invasion of A549 and NCI-H1703 lung cancer cells. This effect is associated with increased expression of N-cadherin, vimentin, MMP-9, and MMP-1, alongside reduced expression of E-cadherin. miR-210-3p further inhibits FGFRL1, promoting EMT and enhancing tumor migration and invasion (132).

Several miRNAs also act as negative regulators of lung cancer progression through diverse mechanisms. For instance, miR-124-3p, miR-326, miR-15a-5p, and miR-338-3p have been identified as inhibitors of lung cancer metastasis. These miRNAs typically bind to the 3’UTR of target mRNAs, leading to either mRNA degradation or inhibition of translation (133). For example, serum exosomal miR-124-3p in NSCLC inhibits exosome secretion and activation of the PI3K/AKT signaling pathway in A549 and NCI-H1299 cells by targeting the 3’UTR of Rab27a. Conversely, LINC00511 functions as a competing endogenous RNA for miR-124-3p, counteracting its inhibitory effects and promoting NSCLC metastasis (55). Similarly, miR-130b-3p, found in the serum exosomes of patients with NSCLC, reduces DEPDC1 expression by directly binding to its 3’UTR, which promotes apoptosis through the TGF-β signaling pathway and inhibits EMT and metastasis in NSCLC cells (134).

In another example, exosomal miR-3180-3p derived from A549 cells suppresses proliferation and metastasis by targeting the oncogene FOXP4 in NSCLC cells (135). Additionally, studies have shown that apelin, a natural ligand for G protein-coupled receptors, can promote tumor growth as an oncogenic factor in various cancers (136). However, miR-15a-5p, which is underexpressed in lung cancer tissues, negatively regulates the proliferation and migration of A549 lung cancer cells by targeting CDCA4. It also modulates EMT by increasing E-cadherin expression and regulating vimentin and N-cadherin levels (137). Furthermore, exosomal miR-338-3p has been shown to inhibit the growth and metastasis of A549 and SK-MES-1 cells by targeting CHL1 and influencing the MAPK signaling pathway (138).

lncRNAs, circRNAs, and proteins in exosomes also contribute to lung cancer metastasis and invasion through various pathways and signaling axes. For example, upregulation of exosomal lncRNA ZBED5-AS1 in LUAD-derived HCC827 cells increases the growth, migration, and invasiveness of H1299 and A549 cells by activating the ZNF146/ATR/CHK1 signaling axis and promoting EMT (139). On the other hand, exosomal lncRNA FGD5-AS1 from A549 cells decreases the viability, migration, and invasion of A549 and H1299 cells. In NSCLC tissues, lncRNA FGD5-AS1 targets miR-944, and its knockdown reduces the viability, migration, and invasion of NSCLC cells, thereby attenuating metastasis (140). Additionally, exosomal HOTAIR, derived from NCI-H1975 cells, has been closely linked to lymph node metastasis and TNM clinical staging, enhancing the proliferation and migration of A549 cells. Similarly, LINC00963 in A549 cell exosomes stabilizes the Zeb1 protein by interacting with HNRNPA2B1, facilitating EMT and promoting proliferation, migration, and invasion in A549 and NCI-H1650 cells (141).

Finally, human serum exosomal circSATB2, which is highly expressed in A549, H460, and H1299 cells, upregulates fascin homologous protein 1 (FSCN1) in lung tumor cells by targeting miR-326. Exosomal delivery of FSCN1 enhances NSCLC cell growth, migration, and invasion (142).

Research has demonstrated that circ_0008717 is highly expressed in NSCLC cells, such as A549 and H1299, and functions as an oncogenic factor within exosomes by binding to miR-1287-5p, leading to increased PAK2 expression and accelerated NSCLC tumor formation (143). Additionally, exosomes derived from A549 and H1299 cells containing circRAPGEF5 were found to enhance proliferation, migration, invasion, and EMT in lung adenocarcinoma cells (HCC827, A549, and H1299). In lung adenocarcinoma, ZEB1, negatively regulated by miR-1236-3p, has been identified as a critical target for circRAPGEF5. The inhibition of miR-1236-3p reversed the decrease in ZEB1 expression caused by circRAPGEF5 knockdown, highlighting circRAPGEF5’s significant role in cell proliferation and metastasis through modulation of the miR-1236-3p/ZEB1 signaling pathway (144).

Moreover, Leucine-rich α2-glycoprotein-1 (LRG1), which is highly expressed in A549 and PC-9 cells, has been shown to enhance proliferation, migration, invasion, and EMT of NSCLC cells. Research indicates that exosomal LRG1, abundant in NSCLC cells, promotes tumor metastasis in animal models (145).

The biologically active substances within exosomes—miRNAs, lncRNAs, circRNAs, and proteins—derived from various cells, play diverse roles in regulating the metastatic and invasive potential of lung cancer. These molecules target specific proteins and genes and modulate key signaling pathways. They can promote lung cancer progression by stimulating proliferation, migration, invasion, and angiogenesis, while others exert a suppressive effect on metastasis. This dual role suggests that targeting the pro-metastatic proteins and pathways while enhancing those that inhibit metastasis may be a viable strategy to reduce lung cancer spread, ultimately improving patient survival rates and outcomes.

## 4 Clinical application of exosomes as biomarkers for lung cancer

Interest in exosomes as biomarkers and therapeutic targets is rapidly increasing, particularly in oncology (146). Exosomes serve as versatile carriers, capable of being engineered to improve sampling and targeting precision. Their key benefits include low immunogenicity, minimal toxicity, and high stability (147). The miRNAs, mRNAs, and proteins encapsulated in exosomes are highly stable, making them promising candidates for predicting the health trajectory of patients with malignant tumors (148–151). Additionally, elevated levels of lncRNA have been closely associated with the regulation of tumor behavior across various cancers, including NSCLC (152).

Liquid biopsy has emerged as a highly promising, non-invasive approach for lung cancer screening (153). Among the techniques used, exosome testing has gained popularity within liquid biopsy (29, 154) in recent years due to its substantial advantages in predicting cancer progression and metastasis (29). The poor prognosis of patients with patients with NSCLCs, largely driven by high recurrence rates, underscores the critical need for early identification of novel, sensitive, and specific biomarkers (155). Conventional lung cancer markers, such as carcinoembryonic antigen (CEA), neuron-specific enolase (NSE), cytokeratin 19 fragment (CYFRA21-1), and squamous cell carcinoma antigen (SCCA), are limited by their low sensitivity and specificity (156). In contrast, exosomes offer a more reliable platform for diagnosing lung cancer, monitoring patient survival, evaluating metastasis, and improving diagnostic accuracy.

### 4.1 Diagnosis of lung cancer

Distinguishing between healthy individuals and patients can be effectively achieved by analyzing differences in exosome expression levels *in vivo*. For instance, both patients with NSCLC and those with early-stage NSCLC exhibited significantly reduced miR-620 levels in their exosomes compared to healthy individuals. These differences yielded area under the curve (AUC) values of 0.728 and 0.707, respectively, demonstrating relatively high diagnostic accuracy (157). Moreover, exosome levels in bodily fluids, such as blood and urine, offer additional diagnostic insights. Patients with NSCLC, for example, show significantly elevated expression of DLX6-AS1 in their blood (158). Serum exosomal lncRNAs TBILA and AGAP2-AS1 were also highly expressed in patients with NSCLC, with critical diagnostic values of 0.923 and 1.12, respectively, distinguishing patients from healthy cohorts (159). Additionally, urinary exosomal markers, such as WASL, which is downregulated in patients with lung cancer, and STK10 and WNK1, which are upregulated, can aid in diagnosis. These markers, when detected together in urinary exosomes, yield an AUC value of 0.760 for lung cancer detection (160).

### 4.2 Assessment of patient survival duration

Exosome expression also provides insights into patient survival outcomes. For instance, a combination of biomarkers, including the protein levels of fibrinogen beta chain, fibrinogen gamma chain, and von Willebrand factor, has shown strong potential for diagnosing early-stage NSCLC and correlates directly with patient survival (161). Lower levels of BTG-1 in plasma exosomes were associated with shorter disease-free and overall survival times compared to higher levels (162). Similarly, high miR-125b-5p expression in lung cancer tissues was linked to better overall survival, while low expression correlated with worse outcomes (155). Exosomal levels of miR-5684 and miR-125b-5p were lower in patients with NSCLC than in healthy individuals, with an AUC value of 0.793. Additionally, lncRNA-SOX2OT expression in exosomes from patients with NSCLC showed a negative correlation with overall survival, making it a valuable prognostic marker (65). These findings highlight the utility of these exosome-derived biomarkers as diagnostic and prognostic tools in NSCLC.

### 4.3 Assessing the metastatic capacity of lung cancer

The metastatic potential of lung cancer can also be evaluated based on exosome expression and AUC values. For instance, miR-1290 expression in exosomes can differentiate between patients with LUAD at various stages (163). LUCAT1 promotes metastasis in LUAD cells by adsorbing miR-4316, thereby releasing VEGFA (155). Biomarkers such as CFHR5, C9, and MBL2, particularly CFHR5 alone, are significantly associated with overall survival in patients with NSCLC (161). Furthermore, lipopolysaccharide-binding protein has demonstrated the ability to distinguish between metastatic and non-metastatic NSCLC cases, with an AUC value of 0.803, further underscoring its efficacy as a marker of metastatic potential (164).

### 4.4 Improving detection accuracy

The reliability of commonly tested tumor markers, such as CEA, CYFRA21-1, NSE, and SCCA, is limited due to issues with sensitivity and specificity (156). To improve diagnostic accuracy, alternative markers and combination approaches are often utilized. Serum-derived exosomes, secreted by tumor cells, offer a more precise and dynamic reflection of tumor cell activity compared to tissue-based assessments. Detecting miRNAs in exosomes allows for real-time monitoring through blood samples, eliminating the spatial heterogeneity found in tissue biopsies and providing a more continuous assessment of treatment response. Furthermore, the dual membrane structure of exosomes ensures the stability of internal miRNAs in the bloodstream, offering higher reliability for detection (157). For example, serum exosomal miR-216b has been shown to effectively differentiate between patients with NSCLC and healthy controls (165).

It is well established that versican is notably upregulated in the plasma and plasma exosomes of patients with NSCLC. Receiver operating characteristic analysis indicated that the AUC values of versican in both plasma and exosomes exceeded those of traditional markers, such as NSE, CYFRA21-1, and SCCA, indicating superior diagnostic efficacy in NSCLC (166). Similarly, plasma exosomal BTG-1 more accurately predicted three-year disease-free and overall survival in patients with NSCLC, demonstrating stronger discrimination and specificity (162). The combined use of miRNAs has also been shown to enhance diagnostic accuracy for SCLC, with a 3-miRNA linear combination model (miR-200b-3p, miR-3124-5p, and miR-92b-5p) demonstrating a significant association with lung disease presence, including lung cancer (167). Moreover, integrating exosomal markers with traditional tumor markers further improved diagnostic performance. While combining lncRNAs did not outperform individual ones, pairing exosomal TBILA and AGAP2-AS1 with Cyfra21-1 resulted in improved accuracy for NSCLC diagnosis (159). Likewise, serum exosomal miR-216b significantly enhanced diagnostic performance when combined with standard tumor markers (165). Evaluating exosomal miR-1290 and lncRNA DLX6-AS1 alongside conventional markers further improved sensitivity and specificity in comparison to standard markers alone (158, 163).

Thus, lung cancer diagnosis, prognosis, metastasis evaluation, and test precision can be significantly enhanced by examining exosome expression *in vivo*. The levels of exosomal biomarkers, their AUC values, and their inherent stability play pivotal roles in diagnostic accuracy. Notably, the integration of exosomes with traditional tumor markers has shown promise in enhancing both the precision and specificity of lung cancer diagnostics and treatment strategies. This combined approach not only offers innovative methodologies for tumor detection but also opens up new avenues for improving the sensitivity and accuracy of cancer diagnosis.

## 5 Conclusion

The role of exosomes in lung cancer metastasis has been extensively explored, highlighting their critical involvement in communication between tumor cells. Through the regulation of exosomal content, cancer cells can manipulate the microenvironment of distant organs, facilitating metastasis and influencing organ-specific preferences, thus accelerating the spread of cancer.

This review presents a comprehensive overview of the multiple mechanisms by which exosomes contribute to the dissemination of lung cancer to the brain and bone, offering potential targets for intervention in both diagnosis and treatment of lung cancer metastasis, a life-threatening condition. Moreover, cancers such as breast, bone, and colon utilize exosomes to influence the formation of PMNs before metastasizing to the lungs, pointing to a possible strategy for preventing lung metastasis. This underscores the critical role of PMNs in both lung cancer metastasis to other organs and the metastasis of cancers from other sites to the lungs, all facilitated by exosomes. These processes involve the establishment of a favorable microenvironment for tumor metastasis, achieved through various mechanisms that are difficult to detect in clinical settings. Therefore, monitoring the establishment of such environments by tumor cells and identifying indicators that signal a shift toward metastasis are key to deepening our understanding of exosome-mediated metastasis.

In addition, the process of lung metastasis is heavily influenced by angiogenesis and macrophage polarization toward the M2 phenotype, both of which are mediated by bioactive substances within exosomes. These substances affect several tumor progression factors, including proliferation, migration, invasion, and ultimately metastasis. Investigating how bioactive molecules from exosomes influence lung cancer metastasis can help identify pathways to enhance anti-metastatic effects or inhibit pro-metastatic signaling. It is essential to recognize that different cell types release exosomes containing distinct bioactive substances, which may act through varying mechanistic pathways. A key consideration in clinical applications is the selection of optimal bioactive substances within exosomes, informed by current research.

Despite the progress in understanding lung cancer metastasis and exosomes, several areas require further investigation. Specifically, the exact mechanisms governing exosome secretion by tumor or immune cells and their role in lung cancer metastasis remain unclear. Additionally, the therapeutic potential of exosomes—including their capacity to enhance cancer treatment by serving as carriers for anticancer agents—requires further elucidation. Another important aspect to explore is whether the diagnostic accuracy of exosomes changes before and after cancer treatment, particularly concerning the use of therapeutic agents. These unresolved questions emphasize the need for more in-depth research and discussion. In conclusion, exosomes hold significant promise in advancing cancer therapy, and future comprehensive studies are both essential and urgent.
